# ECG Prediction Based on Classification via Neural Networks and Linguistic Fuzzy Logic Forecaster

**DOI:** 10.1155/2015/205749

**Published:** 2015-06-29

**Authors:** Eva Volna, Martin Kotyrba, Hashim Habiballa

**Affiliations:** University of Ostrava, 30 Dubna 22, 70103 Ostrava, Czech Republic

## Abstract

The paper deals with ECG prediction based on neural networks classification of different types of time courses of ECG signals. The main objective is to recognise normal cycles and arrhythmias and perform further diagnosis. We proposed two detection systems that have been created with usage of neural networks. The experimental part makes it possible to load ECG signals, preprocess them, and classify them into given classes. Outputs from the classifiers carry a predictive character. All experimental results from both of the proposed classifiers are mutually compared in the conclusion. We also experimented with the new method of time series transparent prediction based on fuzzy transform with linguistic IF-THEN rules. Preliminary results show interesting results based on the unique capability of this approach bringing natural language interpretation of particular prediction, that is, the properties of time series.

## 1. Background

Biometrical data is typically represented as an image or a quantification of measured physiological or behavioural characteristics. As this data should refer to very complex human behaviour or describe very precisely physiological characteristic (typically iris scan, fingerprint, palm vein image, hand scan, voice, walk pattern, etc.), this data can easily become very large and hard to process. For this reason, modern ways of data processing and classification are applied for biometrical data. The leading method is the usage of neural networks [1].

For more than four decades, computers have been used in the classification of the electrocardiogram (ECG) resulting in a huge variety of techniques [2] all designed to enhance the classification accuracy to levels comparable to that of a “gold standard” of expert cardiology opinion. Included in these techniques are multivariate statistics, decision trees, fuzzy logic, expert systems, and hybrid approaches [3]. The recent interest in neural networks coupled with their high levels of performance has resulted in many instances of their application in this field [4].

The electrocardiogram is a technique of recording bioelectric currents generated by the heart. Clinicians can evaluate the conditions of a patient's heart from the ECG and perform further diagnosis. ECG records are obtained by sampling the bioelectric currents sensed by several electrodes, known as leads. A typical one-cycle ECG tracing is shown in Figure 3.

### 1.1. Backpropagation Neural Networks

A neural network is a parallel, distributed information processing structure consisting of processing elements (which can possess a local memory and can carry out localized information processing operations) interconnected together with unidirectional signal channels called connections. Each processing element has a single output connection which branches into as many collateral connections as desired (each carrying the same signal, the processing element output signal). The processing element output signal can be of any mathematical type desired. All of the processing that goes on within each processing element must be completely local: that is, it must depend only upon the current values of the input signals arriving at the processing element via impinging connections and upon values stored in the processing element's local memory [5].

**Figure 1:**
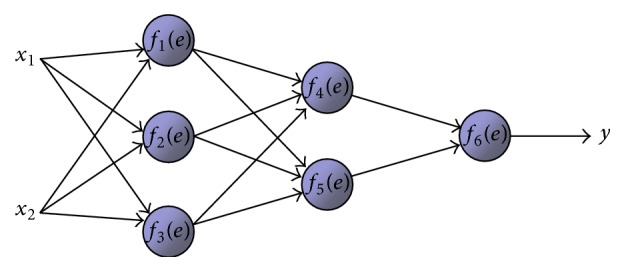
A backpropagation network architecture.

The backpropagation neural network architecture is a hierarchical design consisting of fully interconnected layers or rows of processing units (with each unit itself comprised of several individual processing elements). Backpropagation belongs to the class of mapping neural network architectures and therefore the information processing function that it carries out is the approximation of a bounded mapping or function *f* : *A* ⊂ *R* ^*n*^ → *R* ^*m*^, from a compact subset **A** of* n*-dimensional Euclidean space to a bounded subset *f*[**A**] of* m*-dimensional Euclidean space, by means of training on examples (*x*_1_, *z*_1_), (*x*_2_, *z*_2_),…,(*x*_*k*_, *z*_*k*_)…. It will always be assumed that such examples of a mapping *f* are generated by selecting **x**_**k**_ vectors randomly from **A** in accordance with a fixed probability density function *p*(**x**). The operational use to which the network is to be put after training is also assumed to involve random selections of input vectors **x** in accordance with *p*(**x**). The backpropagation architecture described in this paper is the basic, classical version (Figure 1). The backpropagation learning algorithm is composed of two procedures: (a) forward propagation of signals and (b) backpropagation weight training [5].

*Feed-Forward. *Assume that each input factor in the input layer is denoted by *x*_*i*_; the *y*_*j*_ and *z*_*k*_ represent the output in the hidden layer and the output layer, respectively. And the *y*_*j*_ and *z*_*k*_ can be expressed as follows (1):

(1) $$\begin{array}{c} y_{j}=f\left(X_{j}\right)=f\left(w_{oj}+\overset{I}{\underset{i=1}{\sum }}w_{ij}x_{i}\right),\\ z_{k}=f\left(Y_{k}\right)=f\left(w_{ok}+\overset{J}{\underset{j=1}{\sum }}w_{ik}y_{j}\right), \end{array}$$

where the *w*_*oj*_ and *w*_*ok*_ are the bias weights for setting threshold values, *f* is the activation function used in both hidden and output layers, and *X*_*j*_ and *Y*_*k*_ are the temporarily computing results before applying activation function *f*. In this study, a sigmoid function is selected as the activation function. Therefore, the actual outputs *y*_*j*_ and *z*_*k*_ in hidden and output layers, respectively, can be also written as

(2) $$\begin{array}{c} y_{j}=f\left(X_{j}\right)=\frac{1}{1+e^{-X_{j}}},\\ z_{k}=f\left(Y_{k}\right)=\frac{1}{1+e^{-Y_{k}}}. \end{array}$$

The activation function *f* introduces the nonlinear effect to the network and maps the result of computation to a domain (0, 1). This sigmoid function is differentiable. The derivative of the sigmoid function in (2) can be easily derived as *f*′ = *f*(1 + −*f*).

*Backpropagation Weight Training*. The error function is defined as

(3) $$\begin{array}{c} E=\frac{1}{2}\overset{K}{\underset{k=1}{\sum }}e_{k}^{2}=\overset{K}{\underset{k=1}{\sum }}{\left(t_{k}-z_{k}\right)}^{2}, \end{array}$$

where *t*_*k*_ is a predefined network output (or desired output or target value) and *e*_*k*_ is the error in each output node. The goal is to minimize *E* so that the weight in each link is accordingly adjusted and the final output can match the desired output. To get the weight adjustment, the gradient descent strategy is employed. In the link between hidden and output layers, computing the partial derivative of *E* with respect to the weight *w*_*jk*_ produces

(4) $$\begin{array}{c} \frac{\partial E}{\partial w_{jk}}=-e_{k}{f^{\prime }}^{\left(Y_{k}\right)y_{j}}=-\delta_{k}y_{j}\;where\;\;\delta_{k}=\left(t_{k}-z_{k}\right)f^{\prime }\left(Y_{k}\right). \end{array}$$

The weight adjustment in the link between hidden and output layers is computed by Δ*w*_*jk*_ = *α* × *y*_*j*_ × *δ*_*k*_, where *α* is the learning rate, a positive constant between 0 and 1. The new weight herein can be updated by the following *w*_*jk*_(*n* + 1) = *w*_*jk*_(*n*) + Δ*w*_*jk*_(*n*), where *n* is the number of iterations. Similarly, the error gradient in links between input and hidden layers can be obtained by taking the partial derivative with respect to *w*_*ij*_ as

(5) $$\begin{array}{c} \frac{\partial E}{\partial w_{ij}}=-\Delta_{j}x_{j}=f^{\prime }\left(X_{j}\right)\overset{K}{\underset{k=1}{\sum }}\partial_{k}w_{jk}. \end{array}$$

The new weight in the hidden-input links can be now corrected as Δ*w*_*ij*_ = *α* × *x*_*i*_ × Δ_*j*_ and *w*_*ij*_(*n* + 1) = *w*_*ij*_(*n*) + Δ_*j*_. Training the BP-networks with many samples is sometimes a time-consuming task. The learning speed can be improved by introducing the momentum term *η*. Usually, *η* falls in the range 〈0,1〉. For the iteration *n*, the weight change Δ*w* can be expressed. The backpropagation learning algorithm used in artificial neural networks is shown in many text books [3–6].

### 1.2. Fuzzy Logic

Fuzzy logics form heterogeneous family of formalisms capable of successful modelling of uncertain and vague information processing [7]. The usage of fuzzy logic for analysis and prediction of time series can be perceived as a complement method to neural network based methods. The symbolic background of fuzzy logic brings an advantage of human readable symbolic representation of prediction interpretation. It does not necessarily mean that fuzzy logic based time series analysis is more accurate and more efficient but its power lies in transparent and interpretable results that it gives [8–11].

Time series analysis and prediction are an important task that can be used in many areas of practice. The task of getting the best prediction to given series may bring interesting engineering applications in wide number of areas like economics, geography, or industry. Solution to the problem of obtaining best results in prediction of time series can be based on well-known and simple methods like Winters or Linear method. In this paper, we use a method based on two methods originally developed by members of Institute for Research and Applications of Fuzzy Modeling, which is a part of University of Ostrava. The aim of the paper is not to present the details of the methods already published but to present a tool implementing them. The first method is based on the notion of F-transform (fuzzy transform) devised by the group of Professor Perfilieva et al. [12]. The second approach uses the linguistic rules utilizing fuzzy logic and deduction that is a well-known formalism with very good results in variety of practical applications like industrial ones.

The idea of the fuzzy transform is to transform a given function defined in one space into another, usually simpler space, and then to transform it back. The simpler space consists of a finite vector of numbers. The reverse transform then leads to a function, which approximates the original one. More details can be found in [12].

The fuzzy transform is defined with respect to a fuzzy partition, which consists of basic functions. Let *c*_1_ < ⋯<*c*_*n*_ be fixed nodes within [*a*, *b*] such that *c*_1_ = *a*, *c*_*n*_ = *b*, and *n* ≥ 2. We say that fuzzy sets *A*_1_,…, *A*_*n*_ ∈ *F*([*a*, *b*]) are basic functions forming a fuzzy partition of [*a*, *b*] if they fulfill the following conditions for *i* = 1,…, *n*:

(1) *A*_*i*_(*c*_*i*_) = 1;
(2) *A*_*i*_(*x*) = 0 for *x*/∈(*c*_*i*−1_, *c*_*i*+1_), where for uniformity of notation we put *c*_0_ = *c*_1_ = *a* and *c*_*n*+1_ = *c*_*n*_ = *b*;
(3) *A*_*i*_ is continuous;
(4) *A*_*i*_ strictly increases on [*c*_*i*−1_, *c*_*i*_] and strictly decreases on [*c*_*i*_, *c*_*i*+1_];
(5) for all *x* ∈ [*a*, *b*],
  (6) $$\begin{array}{c} \overset{n}{\underset{i=1}{\sum }}A_{i}\left(x\right)=1. \end{array}$$

Let a fuzzy partition of [*a*, *b*] be given by basic functions *A*_1_,…, *A*_*n*_, *n* ≥ 2 and let *f*: [*a*, *b*] → *R* be a function that is known on a set {*x*_1_,…, *x*_*T*_} of points.

The* n*-tuple of real numbers [*F*_1_,…, *F*_*n*_] given by

(7) $$\begin{array}{c} F_{i}=\frac{\sum_{t=1}^{T}f\left(x_{t}\right)A_{i}\left(x_{t}\right)}{\sum_{t=1}^{T}A_{i}\left(x_{t}\right)},\;i=1,\ldots ,n, \end{array}$$

is a fuzzy transform of *f* with respect to the given fuzzy partition.

The numbers *F*_1_,…, *F*_*n*_ are called the components of the fuzzy transform of *f*.

Let *F*_*n*_[*f*] be the fuzzy transform of *f* with respect to *A*_1_,…, *A*_*n*_ ∈ *F*([*a*, *b*]).

Then the function *f*_*F*,*n*_ given on [*a*, *b*] by

(8) $$\begin{array}{c} f_{F,n}\left(x\right)=\overset{n}{\underset{i=1}{\sum }}F_{i}A_{i}\left(x\right) \end{array}$$

is called the inverse fuzzy transform of *f*.

Fuzzy IF-THEN rules can be understood as a specific conditional sentence of natural language of the form IF *X*_1_ is *A*_1_ AND ⋯ AND *X*_*n*_ is *A*_*n*_ THEN *Y* is *B*, where *A*_1_,…, *A*_*n*_ and *B* are evaluative expressions (very small, roughly big, etc.). An example fuzzy IF-THEN rule is as follows.

*IF the number of cars sold in the current year is more or less small and the half-year sales increment is medium, THEN the upcoming half-year increment will be medium*.

The part of the rule before THEN is called the antecedent and the part after it is consequent. Fuzzy IF-THEN rules are usually gathered in a linguistic description:

(9) R1≔IF  X1  is  A11  AND⋯AND  Xn  is  A1n  THEN  Y  is  B1,⋮Rm≔IF  X1  is  Am1  AND⋯AND  Xn  is  Amn  THEN  Y  is  Bm.

Time series prediction based on these two main approaches works as follows. Let time series *x*_*t*_, *t* = 1,…, *T* be viewed as a discrete function *x* on a time axis *t*. Then *F*_*n*_[*x*] = [*X*_1_,…, *X*_*n*_] is the fuzzy transform of the function *x* with respect to a given fuzzy partition. The inverse fuzzy transform then serves us as a model of the trend-cycle of a given time series. By subtracting the trend-cycle (inverse fuzzy transform) values from the time series lags, we get pure seasonal components. This is how the fuzzy transform helps us to model and decompose a given time series.

Logical dependencies between components *X*_1_,…, *X*_*n*_ of the fuzzy transform may be described by the fuzzy rules. These rules are generated automatically from the given data and are used for forecasting the next components. Fuzzy transform components as well as their first and second order differences are used as antecedent variables. For forecasting future fuzzy transform components based on the generated fuzzy rules, a special inference method—perception-based logical deduction is used. The seasonal components are forecasted autoregressively. Finally, both forecasted components, trend-cycle and seasonal, are composed together to obtain the forecast of time series lags. These methods are integrated into an implementation, PC application called* linguistic fuzzy logic forecaster (LFLF)*, which enables as to produce linguistic descriptions that describe properties of data treated like a time series.

## 2. Basic Principles of ECG Evaluation

**Figure 2:**
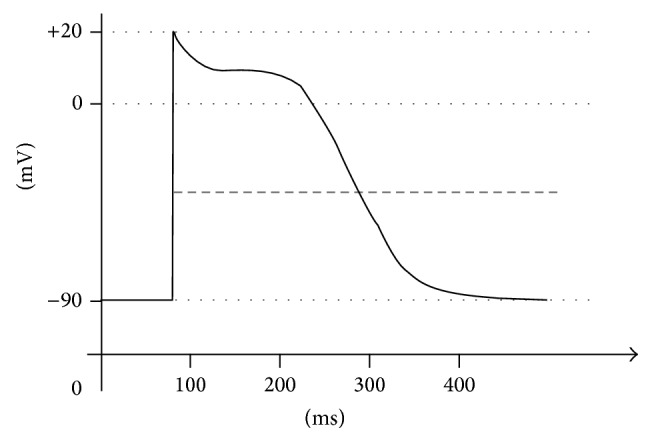
The cardiac action potentials.

ECG scanning has its own rules, which are in accordance with the laws of physics. The heart irritation spreads in all directions. In the case that the depolarisation spreads towards the electrode, which is placed on the body surface, a positive deflection is recorded on an ECG monitor. A negative deflection is recorded at the opposite end of the body. The ECG waveform is written with a chart speed of 25 mm·s^−1^. An algorithm describing the curve goes in the following steps. First, we evaluate the shape and rhythm of ventricular complexes or atrial, which can be either regular or irregular. Then we evaluate the frequency of ventricular complexes and atrial fibrillations. Contraction of each muscle of the human body (and thus the heart as well) is associated with electrical changes called depolarization, which can be detected by electrodes. The heart contains two basic types of cells: myocardial cells, which are responsible for generating the pressure necessary to pump blood throughout the body, and conduction cells, which are responsible for rapidly spreading electrical signals to the myocardial cells in order to coordinate pumping. A graph of an action potential of a muscle of cardiac cells is shown in Figure 2.

**Figure 3:**
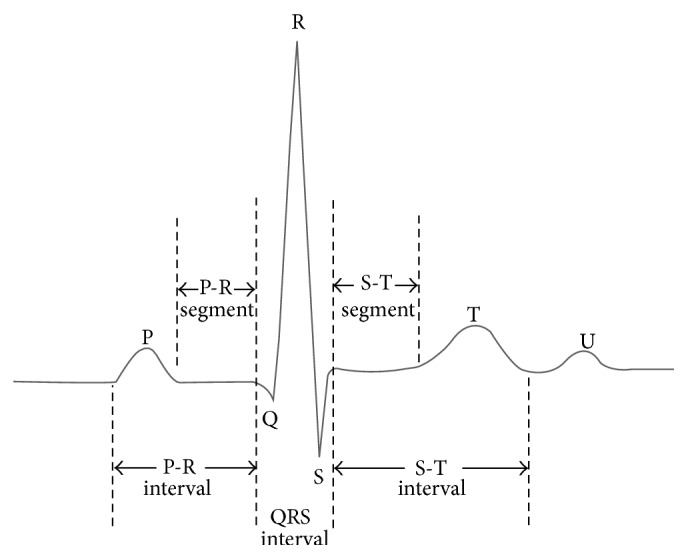
A typical one-cycle ECG tracing (adapted from http://www.ni.com/white-paper/app/largeimage?lang=cs&imageurl=%2Fcms%2Fimages%2Fdevzone%2Ftut%2F2007-07-09_141618.jpg).

A normal electrocardiogram is illustrated in Figure 3. The figure also includes definitions for various segments and intervals in the ECG. The deflections in this signal are denoted in alphabetic order starting with the letter* P*, which represents atrial depolarization. The ventricular depolarization causes the* QRS* complex, and repolarization is responsible for the T-wave. Atrial repolarization occurs during the* QRS* complex and produces such a low signal amplitude that it cannot be seen apart from the normal ECG.

## 3. Signal Processing Using Neural Networks and Fuzzy Logic

In practice, a relatively reliable diagnostic program stored in ECG monitors has been used, which is a guideline for determining the final diagnosis of heart disorders. This program works according to the principle of IF-THEN rules. The values of the electrical signal are discretized and uploaded into expert systems in the form of thousand rules. The aim of this paper is to use a different approach based on the principle of neural networks. The proposed methodology of solution could be summarized into the following steps:

1. a conversion of analog signal from the ECG monitor to a computer,
2. using multilayer networks that are fully connected,
3. obtaining ECG waveforms in collaboration with the University Hospital in Poruba, specifically at the Department Cardiac Surgery from sick patients and at the Department Traumatology from healthy patients (i.e., “healthy” with regard to heart diseases),
4. ECG waveforms built training/test sets,
5. neural network adaptation,
6. testing phases.

### 3.1. Technical Equipment

ECG measurements were performed using ADDA Junior with converter ADDA Junior, which was connected to a computer via bidirectional parallel cable (CETRONICS). Technical parameters of the A/D converter (8-bit conversion) were the following:

- 3 measuring ranges,
- measuring of a frequency of AC voltage at any channel,
- autoranging for measuring the frequency of 100 Hz, 1 kHz, and 10 kHz,
- input resistance of 300 kΩ,
- measurement accuracy 1%.

Technical parameters of the /D/A converter (a programmable voltage source ±10 V) were the following:

- maximum current consumption of 15 mA (after optimizing 4A at the output),
- power of the converter ±15 V (stabilized).

## 4. Experimental Results

### 4.1. Time Series Classification and Prediction via Neural Networks

**Figure 4:**
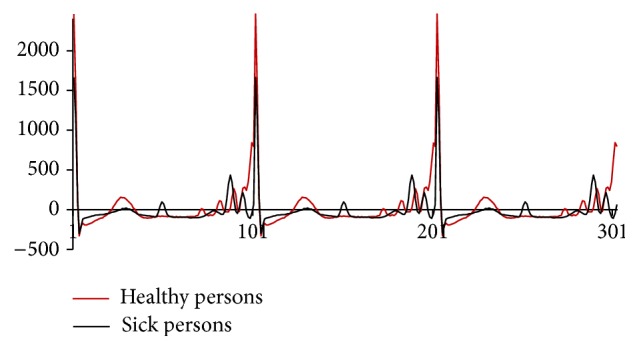
Comparison of mean values of ECG waveforms for healthy/sick persons.

The training set consisted of modified ECG waveforms. We used a backpropagation neural network with topology 101-10-1. The output unit represents a diagnose 0/1, a healthy/sick person. A smaller number of inputs would not be appropriate due to the nature of the ECG waveform. We use 34 ECG time series associated with sick persons and 36 ECG time series associated with healthy persons. 25 time series of each group were used as a training set and the rest as a test set.  Figure 4 shows a comparison of mean values of ECG waveforms for healthy/sick persons. We used the backpropagation method [5, 6] for the adaptation with the following parameters: the learning rate value is 0.1 and momentum is 0. The conducted experimental studies also showed that training patterns are mixed randomly in each cycle of adaptation. This ensures their greater diversity which acts as a measure of system stability. Uniform system in a crisis usually collapses entirely, while system with such diversity of trained patterns remains functional despite of crisis of its individual parts. The condition of end of the adaptation algorithm specified the limit value of the overall network error, *E* < 0.1.

**Figure 5:**
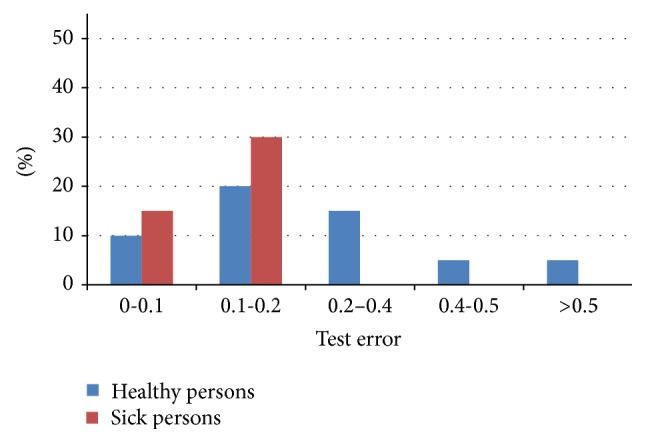
Experimental results, test error for healthy/sick persons.

The test set consisted of 20 samples (11 health and 9 sick persons) that were not included in the training set. The summary results for this type of experiment are shown in a graph in Figure 5. For clarity, the results of testing are given in percentage. The average test error was 0.194. A healthy population was detected with an average error of 0.263 and sick population with an average error of 0.109.

#### 4.1.1. Pattern Recognition Classifier Leading to Prediction

**Figure 6:**
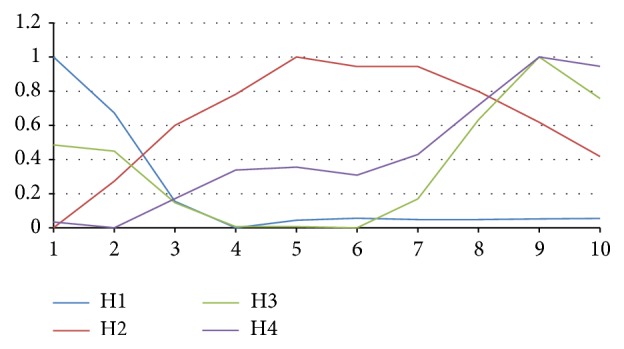
Patterns representing healthy persons.

**Figure 7:**
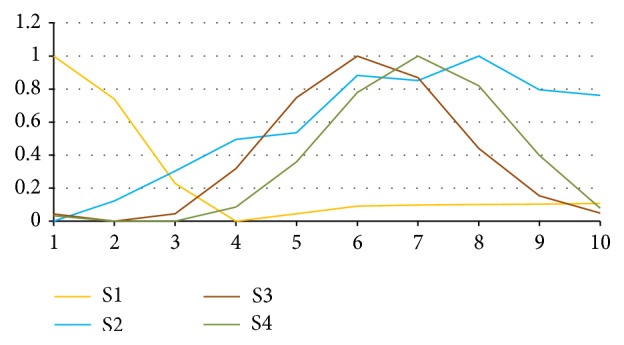
Patterns representing sick persons.

For the purpose of adaptation of the pattern recognition classifier, it is necessary to remark that determination of training patterns is one of the key tasks. Improperly chosen patterns can lead to confusion of neural networks. During our experimental work, we made some study which included ECG pattern recognition. When creating appropriate patterns of the training set, we used characteristic curves shown as mean values from ECG waveforms for healthy and sick persons (Figure 4). We use two different groups of patterns. Patterns H1–H4 (Figure 6) represent healthy persons and patterns S1–S4 (Figure 7) represent sick persons. The whole training set is shown in Table 1.

Pattern recognition classifier is based on backpropagation neural network and is able to recognise wave structures in given time series [13, 14]. Artificial neural networks need training sets for their adaptation. In our experimental work, the training set consisted of 8 patterns representing the basic structure of the various waves in ECG graphs; see Figures 6 and 7. Input data is sequences always including *n* consecutive numbers, which are transformed into interval 〈0,1〉 by formula (10). Samples are adjusted for the needs of backpropagation networks with sigmoid activation function in this way [5, 6]:

(10) xj′=xj−minxi,…,xi+n−1maxxi,…,xi+n−1−minxi,…,xi+n−1,j=i,…,i+n−1,

where *x*_*j*_′ is normalized output value of the *j*th neuron (*j* = *i*,…, *i* + *n* − 1) and (*x*_*i*_,…*x*_*i*+*n*−1_) are *n* − 1 consecutive output values that specify sequences (patterns) from the training set (e.g., training pars of input and corresponding output vectors). Input vector contains 10 components. Output vector has got 8 components and each output unit represents one of 8 different types of ECG wave samples. A neural network architecture is 10-10-8 (e.g., 10 units in the input layer, 10 units in the hidden layer, and 8 units in the output layer). The net is fully connected. Adaptation of the neural network starts with randomly generated weight values.

We used the backpropagation method for the adaptation with the following parameters: the learning rate value is 0.1 and momentum is 0. We have utilized our experience from earlier times; that is, training patterns were mixed randomly in each cycle of adaptation. The condition of end of the adaptation algorithm specified the limit value of the overall network error, *E* < 0.1.

**Figure 8:**
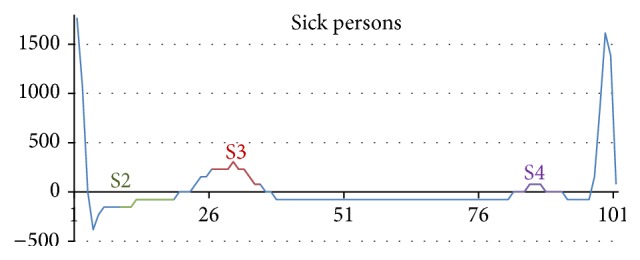
Some recognized patterns that occur in ECG time series.

**Figure 9:**
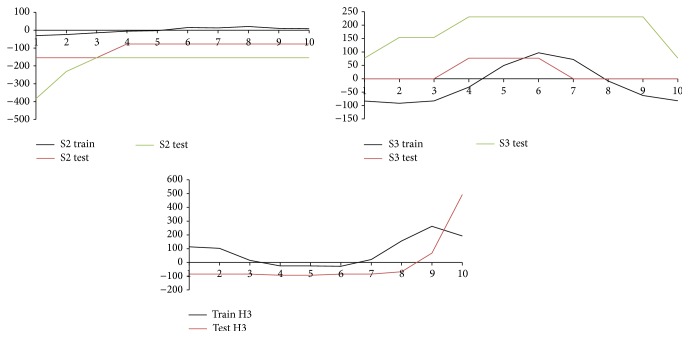
Training patterns, their representation in used test sets.

In order to test the efficiency of the method, we applied the same set of data that we used in the previous experimental part. Outputs from the classifier produce sets of values that are assigned to each recognized training pattern in the given test time series. It is important to appreciate what can be considered as an effective criterion related to consensus of similarity. The proposed threshold resulting from our experimental study was determined at least *p* = 70%.  Figure 9 shows a comparison of patterns, how were learned (S2, S3, H3 train) and how were recognized in test time series (S2, S3, H3 test). The neural network is able to discover some connections, which are almost imperceptible. Illustration of some recognized patterns that occur in ECG time series is shown in Figure 8. Outputs from the classifier carry a predictive character. The neural network determines if the time series belongs to a healthy or sick person on the basis of the recognised ECG patterns which appear in the time series history.

**Figure 10:**
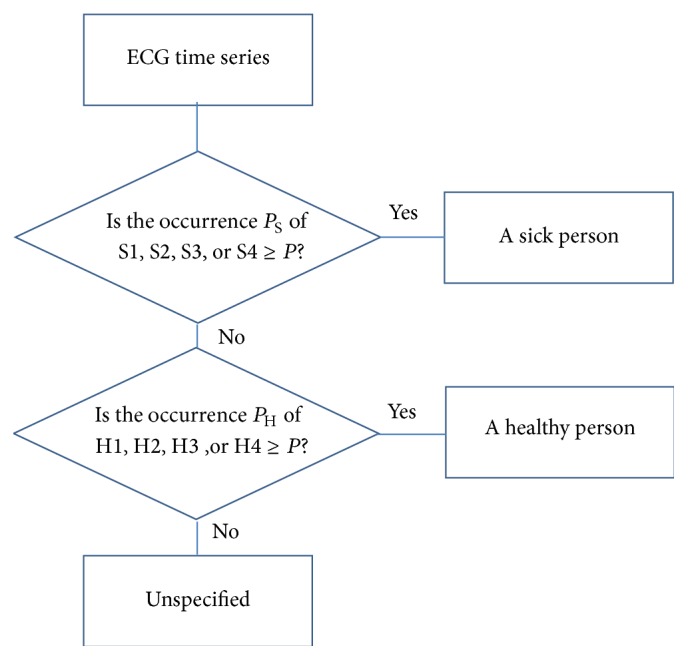
The methodology of testing.

**Figure 11:**
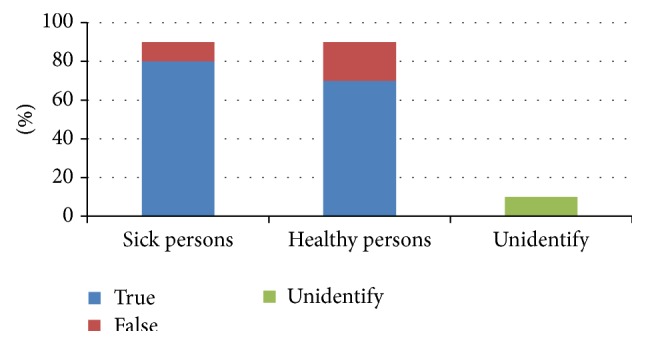
Experimental results, test error.

The methodology of testing is shown in Figure 10. This means that if the test pattern S1, S2, S3, or S4 appeared in ECG waveform with probability *p*_S_ ≥ *p* (*p* = 70%), thus it was predicted to be “a sick person.” Then we work only with the remaining time series. If the test pattern H1, H2, H3, or H4 appeared in ECG waveform with probability *p*_H_ ≥ *p* (*p* = 70%), thus it was predicted to be “a healthy person.” In all other cases, the ECG time series was unspecified. We examined a total of 20 data sets. Each of them contains 101 values that assign 92 possible patterns. The whole number of examined patterns is 1840. The graph in Figure 11 demonstrates a summary of results, where “sick persons” represent patterns S1–S4 and “healthy persons” represent patterns H1–H4. The resulting prediction is based on the methodology; see Figure 10.

### 4.2. Time Series Classification and Prediction via Linguistic Fuzzy Logic Forecaster

**Figure 12:**
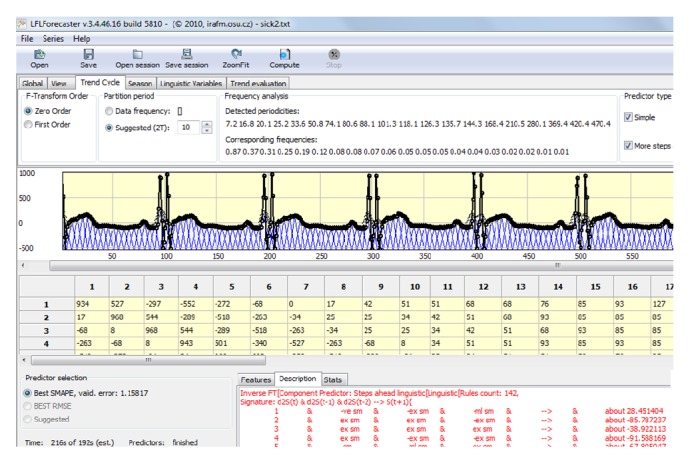
LFLF application.

We tried also to utilize above presented method of time series analysis through linguistic fuzzy logic forecaster (LFLF) [15]; see Figure 12.

Basic usage of the application is to analyse given time series and find best predictor with respect to validation part of time series given. We evaluate efficiency of predictors by* SMAPE* (symmetric mean absolute percentage error). It enables us to make analysis of trend-cycle of a time series and also seasonal part. The main advantage lies in prediction based on transparent linguistic descriptions that provide the model of a time series behaviour. Linguistic variables are of the following types:

- value: we directly mean the components of the fuzzy transform,
- difference, first order differences of fuzzy transform components that are given as follows: differences between components Δ*X*_*i*_ = *X*_*i*_ − *X*_*i*−1_,
- second difference: these are values of second order differences of components of the fuzzy transform as follows: Δ^2^ *X*_*i*_ = Δ*X*_*i*_ − Δ*X*_*i*−1_.

LFLF application enables us to define minimal and maximal number of these particular variables in a rule of linguistic description as well as the total number of antecedent variables.

A rule consisting of these variables has the following structure and can be described as a signature (fuzzy rules describing the trend-cycle model). Particularly, *S* denotes the trend-cycle components, *dS* their differences, and *d*2*S* their second order differences. The argument (*t*), (*t* − 1), and so forth, denotes the time lag of the component.

For example, taking signature *S*(*t*)&*dS*(*t*) → *dS*(*t* + 1) denotes the fact that *X*_*i*_ and Δ*X*_*i*_ are the antecedent variables and Δ*X*_*i*+1_ is the consequent variable of the winning model and hence, we deal with rules of the form

(11) $$\begin{array}{c} IF\;\;X_{i}\;\;is\;\;A_{i}\;\;AND\;\;\Delta X_{i}\;\;is\;\;A_{\Delta i}\;\;THEN\;\;\Delta X_{i+1}\;\;is\;\;A_{Deltai}. \end{array}$$

**Figure 13:**
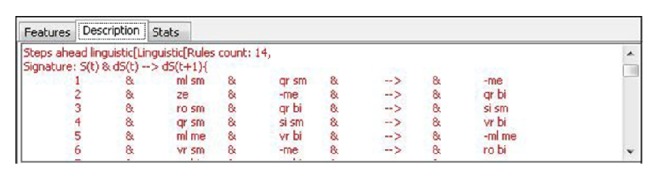
Winning predictor linguistic description (trend-cycle model).

Every single fuzzy rule can be taken as a sentence of natural language, for example, first rule from Figure 13.

IF *X*_*i*_ is ml sm AND Δ*X*_*i*_ is qr sm, THEN Δ*X*_*i*+1_ is − me may be read as follows.

If the number of cars sold in the current year is more or less small and the half-year sales increment is quite roughly small then the upcoming half-year increment will be negative medium.

#### 4.2.1. Recognition of “Healthy” and “Sick” Patterns by LFLF

Our method to use linguistic fuzzy logic forecasting is based on simple idea that best predictor learning from both “healthy” and “sick” pattern samples, respectively, can be used for validation with tested pattern taken as validation part of the series. Then we can evaluate SMAPE for both these cases: compound series SMAPE (“healthy” + tested) and SMAPE (“sick” + tested).

*If SMAPE (“healthy” + tested) < SMAPE (“sick” + tested) then the tested pattern is supposed to be “healthy”; otherwise, the tested pattern is supposed to be “sick.”*

**Figure 14:**
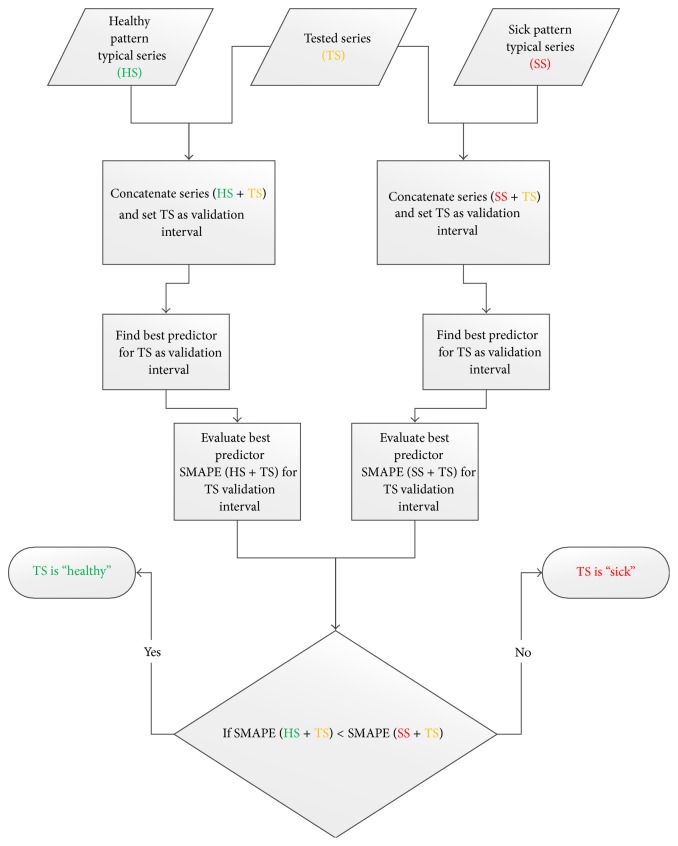
Recognition by linguistic fuzzy logic predictors for typical learning series.

The idea is schematically shown in Figure 14.

For testing purposes, we created two necessary typical learning time series: “healthy” (HS) and “sick” (SS) according to the algorithm above. They both consist of 1010 samples made from 10 typical series of “healthy” and “sick” patients with 101 measured ECG values. Then we have created 10 concatenated series according to the scheme in Figure 14 with 10 randomly selected patients with “healthy” ECG measurement; that is, 20 files were produced (10x HS + TS and 10x SS + TS). The same concatenated series were also made from 10 “sick” patients measurements. This made us additional 20 files with concatenated series (10x HS + TS and 10x SS + TS). For 20 patients (Table 2) tested ECG we have 2 concatenated series giving SMAPE (HS + TS) and SMAPE (SS + TS).

**Figure 15:**
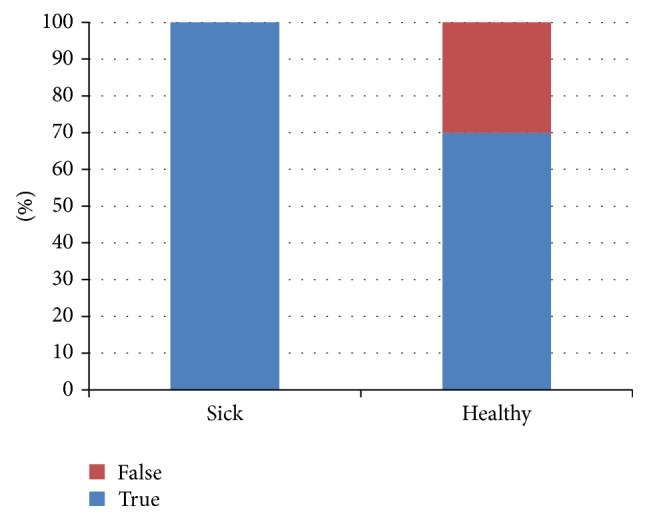
Experimental results, LFLF.

Our method based on LFLF proved very good results for right identification of sick patient records. Nevertheless, it produces large amount of false positive identification of sick pattern for healthy patients (Figure 15). This result is consistent with our approach using neural networks. Of course, our preliminary research has a limited extent and should be perceived only as narrative result, which shows interesting properties especially in complementation of neural network results.

## 5. Conclusion

In this paper, a short introduction into the field of ECG waves recognition using backpropagation neural network has been given. Main objective was to recognise the normal cycles and arrhythmias and perform further diagnosis. We proposed two detection systems that have been created with usage of neural networks. One of them is adapted according to the training set. Here, each pattern represents the whole one ECG cycle. Then, an output unit represents a diagnose 0/1, a healthy/sick person. The second one approach uses neural network, in which training set contains two different groups of patterns for healthy/sick persons. According to the results of experimental studies, it can be stated that ECG waves patterns were successfully extracted in given time series and recognised using suggested method, as can be seen from figures in Experimental Result section. It might result in better mapping of the time series behaviour for better prediction.

Both approaches were able to predict with high probability if the ECG time series represents sick or healthy persons. It is interesting that a sick diagnose was recognised with higher accuracy in both experimental works.

The third approach based on LFLF is currently only in the stage of preliminary experiments, but it conforms to the former results based on neural networks. This approach is novel and could be good supplement to other soft-computing methods for this task.

## Figures and Tables

**Table 1:** The training set.

Patterns	Inputs										Outputs							
H1	1.000	0.672	0.155	0.000	0.045	0.057	0.049	0.049	0.053	0.055	1	0	0	0	0	0	0	0
H2	0.000	0.273	0.600	0.782	1.000	0.945	0.945	0.799	0.618	0.418	0	1	0	0	0	0	0	0
H3	0.485	0.449	0.147	0.007	0.007	0.000	0.169	0.632	1.000	0.757	0	0	1	0	0	0	0	0
H4	0.035	0.000	0.170	0.338	0.356	0.309	0.430	0.719	1.000	0.946	0	0	0	1	0	0	0	0
S1	1.000	0.740	0.228	0.000	0.045	0.091	0.098	0.101	0.104	0.107	0	0	0	0	1	0	0	0
S2	0.000	0.123	0.304	0.495	0.536	0.883	0.851	1.000	0.796	0.761	0	0	0	0	0	1	0	0
S3	0.044	0.000	0.045	0.319	0.748	1.000	0.868	0.440	0.154	0.050	0	0	0	0	0	0	1	0
S4	0.033	0.000	0.000	0.085	0.360	0.779	1.000	0.820	0.399	0.079	0	0	0	0	0	0	0	1

**Table 2:** Example of algorithm evaluation on 10 “healthy” and 10 “sick” patients.

Patient	SMAPE (HS + TS)	SMAPE (SS + TS)	Result	Actual	Match
H11	3.38232	3.30989	Sick	Healthy	**NO**
H12	1.33555	3.40168	Healthy	Healthy	YES
H13	2.73377	4.58243	Healthy	Healthy	YES
H14	3.44581	2.37995	Sick	Healthy	**NO**
H15	2.04677	2.30998	Healthy	Healthy	YES
H16	3.73377	3.98572	Healthy	Healthy	YES
H17	1.03658	2.20151	Healthy	Healthy	YES
H18	3.22689	2.38111	Sick	Healthy	**NO**
H19	2.43544	3.42159	Healthy	Healthy	YES
H20	2.63355	3.79940	Healthy	Healthy	YES
S11	1.72265	1.31922	Sick	Sick	YES
S12	2.04804	0.38562	Sick	Sick	YES
S13	2.99464	0.51305	Sick	Sick	YES
S14	2.52248	0.67941	Sick	Sick	YES
S15	2.65972	1.75233	Sick	Sick	YES
S16	2.63674	2.39694	Sick	Sick	YES
S17	2.57638	1.65941	Sick	Sick	YES
S18	4.24496	2.85006	Sick	Sick	YES
S19	2.96533	1.01009	Sick	Sick	YES
S20	3.23630	1.10960	Sick	Sick	YES
